# Prevalence of *Strongyloides stercoralis* infection and associated clinical symptoms among schoolchildren living in different altitudes of Amhara National Regional State, northwest Ethiopia

**DOI:** 10.1371/journal.pntd.0010299

**Published:** 2022-04-28

**Authors:** Tadesse Hailu Jember, Arancha Amor, Endalkachew Nibret, Abaineh Munshea, Maria Flores-Chavez, Thuy-Huong Ta-Tang, Jose M Saugar, Agustín Benito, Melaku Anegagrie

**Affiliations:** 1 Department of Medical Laboratory Science, College of Medicine and Health Sciences, Bahir Dar University, Bahir Dar, Ethiopia; 2 Mundo Sano Foundation and Institute of Health Carlos III, Madrid, Spain; 3 Biology Department, Science College, Bahir Dar University, Bahir Dar, Ethiopia; 4 Mundo Sano Foundation and National Centre for Microbiology Institute of Health Carlos III, Madrid, Spain; 5 National Center of Tropical Medicine, Institute of Health Carlos III, Biomedical Research Networking Center of Infectious Diseases (CIBERINFEC), Madrid, Spain; 6 National Centre of Microbiology, Institute of Health Carlos III, (CIBERINFEC), Madrid, Spain; 7 National Centre of Tropical Medicine, Institute of Health Carlos III, (CIBERINFEC), Madrid, Spain; The University of Melbourne, AUSTRALIA

## Abstract

**Background:**

*Strongyloides stercoralis* is a parasite that causes strongyloidiasis in humans. It is prevalent in the tropics and sub-tropics where poor sanitation is a common problem. The true prevalence of *S*. *stercoralis* in Ethiopia is underestimated due to the lack of a “Gold” standard diagnostic method. Moreover, its prevalence across altitudinal gradient in Amhara Region has not been studied.

**Methods:**

A cross-sectional study was conducted among 844 schoolchildren in Amhara Region from April to December 2019. A stool sample was collected from each study participant and processed using formol ether concentration technique (FECT), spontaneous tube sedimentation technique (STST), Baermann concentration technique (BCT), agar plate culture (APC) and real-time polymerase chain reaction (RT-PCR). Data were entered using EpiData and analyzed by SPSS version 23 statistical software. Prevalence of *S*. *stercoralis* infection was determined using a single diagnostic technique and combination of techniques. Association of clinical variables with *S*. *stercoralis* infection was assessed by logistic regression and independent variables with p<0.05 were considered statistically significant.

**Results:**

Prevalence of soil-transmitted helminths (STHs) and *S*. *mansoni* infections was 38.0% and 20.4%, respectively. Among STHs, the prevalence of hookworm infection was 32.8%. Prevalence of *S*. *stercoralis* infection was 39.0%, 28.8%, 10.9%, 10.3%, 4.0% and 2.0% by the respective, combinations of the five methods, RT-PCR, APC, BCT, STST and FECT. The highest prevalence rates, 48.2%, 45.0% and 41.1% of *S*. *stercoralis* were recorded in the age group of 12–14 years, males and rural dwellers, respectively. Prevalence rates of *S*. *stercoralis* infection in highland, semi-highland and lowland areas were 40.4%, 41.8% and 25.9%, respectively. Having abdominal pain (AOR = 2.48; 95% CI:1.65–3.72), cough (AOR = 1.63;95%CI:1.09–2.42), urticaria (AOR = 2.49;95%CI:1.50–4.01) and being malnourished (AOR = 1.44;95%:1.10–2.01) were significantly associated with strongyloidiasis.

**Conclusion:**

Prevalence of *S*. *stercoralis* infection was high and varied across different altitudes in Amhara Region. Some clinical syndromes were found to be significantly associated with *S*. *stercoralis* infection. Therefore, proper diagnosis and preventive strategies against *S*. *stercoralis* infection are highly recommended to be devised and implemented in Amhara Region.

## Author summary

Prevalence of *S*. *stercoralis* is under-reported in many resource-poor countries including Ethiopia. We carried out a school-based cross-sectional study among 844 schoolchildren across highlands, semi-highlands and lowland areas of Amhara Region. We used an approach made of five diagnostic methods, namely, FECT, STST, BCT, APC, and RT-PCR. The total prevalence of *S*. *stercoralis* was 39.0%. The highest *S*. *stercoralis* prevalence (41.8%) was observed in the semi-highland areas of the region. Strongyloidiasis was significantly higher among 12–14 years age groups, males, and rural dweller children. Clinical symptoms such as presence of abdominal pain, cough, skin rash and malnourishment were significantly associated with strongyloidiasis. We recommend that preventive strategies against strongyloidiasis in Amhara Region should be implemented.

## Introduction

*Strongyloides stercoralis*, the most important causative agent of strongyloidiasis in human being, is one of the most neglected tropical parasites worldwide [1]. Its prevalence has been increasing in the past few years due to the use of new diagnostic approaches which have increased the detection rate of *S*. *stercoralis* [2]. A global estimate also showed 613.9 million people with strongyloidiasis, of which 76.1% of the infection was accounted by the Southeast Asia, Western Pacific, and African Regions [1]. Even with the existing diagnosis challenges, which are related to the low sensitivity of the available diagnosis methods, and the subsequent under-reporting, the greatest numbers of *S*. *stercoralis* infection are still being noticed from sub-Sahara African countries [3–5].

Strongyloidiasis is an emerging disease in developed countries, where a high number of reports are related to immigrants coming from developing countries [6–7], including immunocompromised individuals [8], travelers and organ transplants recipients [9]. Compared to developed countries, however, *S*. *stercoralis* infection is higher in developing countries. Moreover, prevalence reports coming from these countries are underestimated due to the use of low sensitive diagnostic methods, low larval excretions in the feces, limited knowledge about the infection, and more than 50% of the cases being asymptomatic [10]. The factors listed above make it difficult to get information about the true prevalence of *S*. *stercoralis* infection in developing countries [11].

Since children are frequently playing with soil, they are at risk to be infected by *S*. *stercoralis* [12]. Infected individuals may show skin irritation at the site of skin penetration, a dry cough and/or tracheal irritation, and gastrointestinal symptoms [13]. However, some chronically infected individuals show some mild clinical symptoms including chronic diarrhea, abdominal pain, nausea, and loss of appetite, which are associated with chronic *S*. *stercoralis* infections [14].

Currently, strongyloidiasis faces two challenges for being included in the neglected tropical diseases (NTDs). Firstly, ivermectin (200μg/kg/day for 2 days) and not albendazole is the first line of treatment for the infection [15]. Secondly, the absence of a “Gold” standard diagnostics for strongyloidiasis. Moreover, the employment of other standardized methods which are normally used for detecting helminths’ ova has null sensitivity for detecting *S*. *stercoralis* larvae in soil-transmitted helminths (STHs) endemic areas [16]. For instance, direct saline microscopy is the lowest in its sensitivity, when it is compared with spontaneous tube sedimentation technique (STST), Baermann concentration technique (BCT), agar plate culture (APC) and real-time polymerase chain reaction (RT-PCR) [10]. Baermann concentration technique and APC are the two most suitable parasitological techniques for the diagnosis of *S*. *stercoralis*. Nowadays, PCR techniques are promising [16]. However, these techniques have good specificity but not enough sensitivity; because of that, a combination of techniques is necessary for increasing the detection rate. Unfortunately, the use of a combination of methods as a routine diagnosis approach in endemic countries is limited. As a result, under-diagnosing and under-reporting of *S*. *stercoralis* infection is a common phenomenon.

According to some reports from Amhara Region, Ethiopia, prevalence of *S*. *stercoralis* infection is relatively high [7,17]. Nevertheless, in order to plan control programs, the true prevalence of *S*. *stercoralis* infection and associated clinical symptoms are not known yet in the region. Also, information about the distribution of *S*. *stercoralis* across highlands, semi-highlands, and lowlands areas of Amhara Region is limited. Therefore, this study aimed to determine the prevalence of *S*. *stercoralis* infection using a comprehensive laboratory approach and to identify associated clinical symptoms among schoolchildren across different altitudes in the region.

## Methods and materials

### Ethics statement

Ethical approval was obtained from the Ethical Review Committee of Science College, Bahir Dar University (Ref. No: PGRCSVD/149/2011). Permission letters were obtained from the Amhara National Regional Health Bureau. Supportive letters were also secured from Amhara National Regional Education Bureau, Zonal and Woreda Education Offices. Permits for exporting DNA for molecular analysis in Spain were obtained from the Ethiopian Biodiversity Institute in Addis Ababa (Ref. No: EBI71/1769/2020). Written informed consent was obtained from the parents/guardians of schoolchildren. Study participants who were positive for any of the intestinal parasites were linked to nearby health centers for treatment.

### Study design, area and period

**Fig 1:**
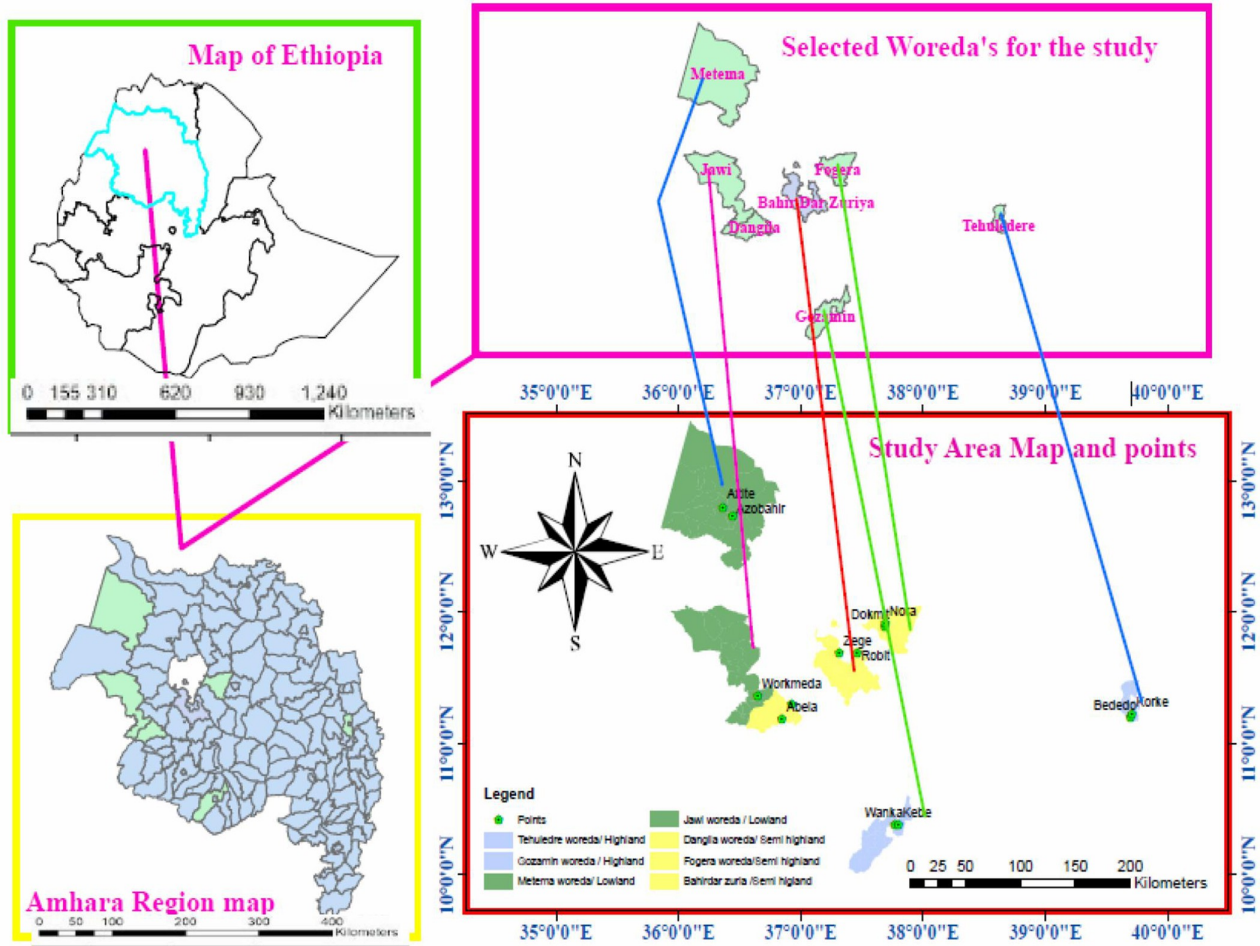
Map of Amhara National Regional State, 2021. A. The location of Amhara National Regional State in Ethiopia, B. The location of selected districts in Amhara National Regional State. C. The site of selected districts. D. The location of primary schools in the selected districts (http://www.planiglobe.com/?lang=enl).

**Fig 2:**
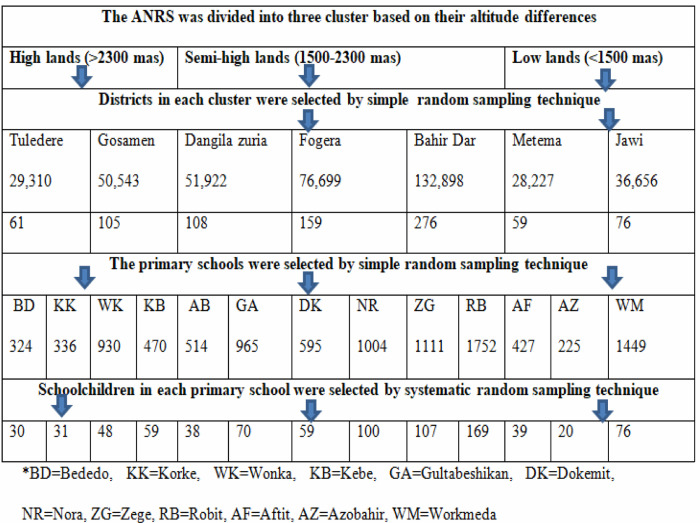
Frame for study sites, schools and schoolchildren in Amhara Region, 2019. The blue arrows show how multi-stage sampling was conducted to select the districts, the primary schools and schoolchildren.

A school-based cross-sectional study was conducted among schoolchildren in Amhara Region from April to December 2019 to determine the prevalence of *S*. *stercoralis* infection across an altitudinal gradient. The annual mean temperature of the region lies between 15°C—21°C. The average annual rainfall is 1145 mm. The wettest and the driest times in Amhara Region are from June to August and October to May, respectively. The major soil types in the region are black (rich in lime, iron, magnesia and alumina), clay, sandy, and silt soils. The Amhara Region is divided into three climatic zones (highland, semi-highland and lowland) based on their altitude difference (Figs 1 and 2).

### Sample size and sampling technique

Since there was no previous *S*. *stercoralis* prevalence data conducted at a regional level, the sample size was calculated by taking P *=* 50%, 95% confidence interval (CI), 5% (d = 0.05) margin of error, and design effect.


$$n=\frac{Z^{2}.P(1-P)}{D^{2}}=\frac{{\left(1.96\right)}^{2}.5(1-0.5)}{0.05^{2}}=384$$

By including 10% of the non-response rate and multiplied by two design effects, the sample size became 844.

Amhara Region was classified into highlands (>2,300 m), semi-highlands (1500-2300m), and lowlands (<1500 m) above sea level by using cluster sampling technique. Two, three and two districts were randomly selected from lowlands, semi-highlands, and highlands areas, respectively. A total of 13 primary schools in seven districts were selected by a simple random sampling technique. Schoolchildren aged 6–14 years and whose parents volunteered to give consent were included in the study. Schoolchildren who had taken anthelmintic drugs in the last three months prior to the data collection period were excluded from the study. The number of study subjects in each primary school was proportionally allocated based on the number of schoolchildren in the school and then children participating in the study were selected by systematic random sampling (Fig 2).

### Data collection

Demographic data were collected from parents using questionnaires whereas clinical information and physical examinations from study subjects were collected by trained health officers.

### Laboratory sample collection and analysis

Intestinal parasites can be detected using a microscope from a stool sample. A fresh stool sample was collected from each study participant using a stool cup and transported to the nearby health institution to detect *S*. *stercoralis* larvae using FECT, STST, BCT, APC and RT-PCR diagnostic tests. The combined result of the five techniques was used to report the *S*. *stercoralis* prevalence.

For FECT, about a half gram of stool was processed in a concentration device based on a modification of Ritchie’s method by Young *et al*., (1979) [18] (BioParaprep-Leti Diagnostics, Barcelona, Spain) which is made up of a collection tube, a filtration unit and a concentration conical tube. Two and half milliliter (mL) of 10% formalin and one mL of ethyl acetate were added into the collection tube. The filtration and the concentration unit were screwed in the top and well closed. The sample was mixed up carefully; the collection tube turned over and spun at 1000 rpm for three minutes. The supernatant in the concentration tube was discarded and a small amount of the sediment was put on a slide, covered with a cover slide and examined for *S*. *stercoralis* larvae using a microscope [18].

For the STST, three grams of stool samples were weighed and homogenized in 10 ml of saline solution. The mixture was filtered through surgical gauze into a 50 ml falcon tube, which was then filled with more saline solution, plugged, and shaken vigorously. The tube was left to stand for 45 minutes. The supernatant was decanted and a sample was taken from the bottom, put on a slide, covered with a cover slide, and examined for *S*. *stercoralis* larvae using microscopy [19].

For BCT, approximately 10–15 grams of fresh, stool sample were weighed and mixed with water and powdered charcoal. The mixture was transferred to a petri dish covered with a double layer of paper towels. Then the mixture was covered with a single layer of paper towel and incubated for 24 hours at 26°C. The incubated stool sample was suspended for one hour in a funnel containing warm water and a sieve and connected to a rubber tube. The filtrate was collected with a tube and centrifuged for five minutes at 2000 rpm. The sediment was mixed, transferred to a slide, and observed with a microscope at 4×, 10× and 40× objectives to detect *S*. *stercoralis* larvae [20].

For the APC, about three grams of feces were placed on the center of the APC media (composed of beef extract, peptone, sodium chloride, agar powder and distilled water) in a Petri dish, sealed with adhesive tape and incubated at 26°C for 48 hours. The surface of the agar plate was analyzed daily with a dissection microscope or with the naked eye for the presence of furrows/tracks of moving larvae [21]. Then, the adhesive tape was removed and five mL of 10% formalin were added to the agar plate surface. After waiting for five minutes, the formalin suspension from the APC was transferred to a test tube and centrifuged at 1500 rpm for five minutes. The sediment was observed for *S*. *stercoralis* and distinguished from hookworm larvae using a microscope with the help of a diagnostic chart [22]. In rhabditiform (L1 and L2) larvae of *S*. *stercoralis* larvae, the rhomboid genital primordium is visible and the buccal cavity is short. However in L1 and L2 hookworm species, the genital primordium is inconspicuous and the buccal cavity is long. Filariform larva (L3) of *S*. *stercoralis* are more slender, have a much longer esophagus than hookworm, and *S*. *stercoralis* has notched tail while hookworm has a pointed tail [23].

For RT-PCR, 180 to 200 mg of the stool sample were used for DNA extraction, which was done with the QIAamp DNA stool mini-kit (Qiagen, Hilden, Germany), following the manufacturer’s instructions. The DNA extraction was performed in Bahir Dar University, College of Medicine and Health Sciences Microbiology laboratory, Bahir Dar, Ethiopia, while the amplification was carried out in Institute of Health Carlos III, Madrid, Spain. Amplification of *S*. *stercoralis* 18S ribosomal ribonucleic acid small subunit was done using specific primers (Forward primer: 5′-GAA TTC CAA GTA AAC GTA AGT CAT TAG C-3′; Reverse primer: 5′-TGC CTC TGG ATA TTG CTC AGT TC-3′) [24]. The final volume of reaction mixture was 25 μl, made of 12.5 μl of QUANTIMIX EASY kit (Biotools, Madrid), 0.5 μl of each forward and reverse primers, 6.35 μl of sterile water and 5 μl of DNA. Two positive controls (a patient diagnosed as positive for *S*. *stercoralis* and stool samples artificially infected with different amounts of *S*. *venezuelensis* L3 DNA), one negative control (a sample from a patient without epidemiological exposure to the parasite and diagnosed as negative for *S*. *stercoralis*) and one blank (distilled water without DNA template) were included. The thermal cycler was programed for an initial denaturation step, run at 95°C for 15 minutes, followed by 50 cycles which included denaturation at 90°C for 10 seconds, annealing at 60°C for 10 seconds, extension at 72°C for 30 seconds; then final extension at 70°C for 10 minutes. Amplification and fluorescence detection for *S*. *stercoralis* real-time PCR was performed on a Corbett Rotor-Gene 6000 RT-PCR cycler (QIAGEN, Hilden, Germany). After completing the RT-PCR run, the specificity of the amplification products was assessed by melting curve analysis and analysis was done with a Rotor Gene 6000 Series software version 1.7 [15,25].

A participant was classified as positive when at least one of the tests was positive for *S*. *stercoralis*.

### Data quality assurance

Prior to data collection, training was given to laboratory personnel and health officers. Stool cups were properly labeled, and the amount of stool in each sample was checked during sample collection. The sample was transported without any preservation method to the nearby health institution laboratory as soon as possible; samples for DNA extraction were stored at -80°C. A standard operating procedure was followed in each laboratory test. To eliminate observer bias, stool slides were examined independently by two laboratory technologists and the results of their observations were recorded on separate sheets for later comparison. The discordant results were re-checked. Generally, data quality was checked during pre-analytical, analytical and post-analytical phases.

### Data analysis

Data were entered into EpiData software and analyzed by using Statistical Package for Social Sciences (SPSS) version 23 statistical software. The overall prevalence of *S*. *stercoralis* infection and the distribution of strongyloidiasis across different altitudes were calculated using descriptive statistics and Chi-square. The strength of association of clinical variables with *S*. *stercoralis* infection was evaluated by Crude Odds Ratio (COR) in a univariate logistic regression model. And then, variables with *p<*0.25 in univariate analysis were selected and entered into a multivariate logistic regression model so as to compute Adjusted Odds Ratio (AOR) and identify the major explanatory variables of the infection among the studied population [26]. Clinical variables with *p<*0.05 in the final model were considered statistically significant.

## Results

### Socio-demographic characteristics of the study participants

A total of 844 schoolchildren participated in this study. The mean age of the study participants was 10.3 years with a standard deviation of 1.77 years ranging from six to 14 years. The majority 364 (43.1%) of study participants were in the age of 10-11years followed by 6–9 age groups 254 (30.1%). The number of male participants was 436 (51.7%). The majority 745 (88.3%) and 765 (90.6%) of the study participants were rural dwellers and Orthodox Christian Church followers, respectively (Table 1). The number of schoolchildren included in the highlands, semi-highlands and lowlands areas were 166 (19.7%), 543 (64.3%), and 135 (16%), respectively (Table 1).

**Table 1:** Socio-demographic characteristics of schoolchildren with their *S*. *stercoralis* and intestinal parasitic infections in Amhara Region, 2019.

Characteristics		Total examined [N,%]	*S*. *stercoralis*		IPIs status	
			Pos [N,%]	Neg [N,%]	Pos [N,%]	Neg [N,%]
**Age (year)**	**6–9**	254 (30.1)	74 (29.1)	180 (70.9)	193 (76.0)	61 (24.0)
	**10–11**	364 (43.1)	146 (40.1)	218 (59.9)	284 (78.0)	80 (22.0)
	**12–14**	226 (26.8)	109 (48.2)	117 (51.8)	196 (86.7)	30 (13.3)
**Gender**	**M**	436 (51.7)	196 (45.0)	240 (55.0)	370 (84.9)	66 (15.1)
	**F**	408 (48.3)	133 (32.6)	275 (67.4)	303 (74.3)	105 (25.7)
**Residence**	**Rural**	745 (88.3)	306 (41.1)	439 (58.9)	601 (80.7)	144 (19.3)
	**Urban**	99 (11.7)	23 (23.2)	76 (76.8)	72 (72.7)	27 (27.3)
**Religion**	**Christian**	765 (90.6)	300 (39.3)	465 (60.8)	620 (81.0)	145 (19.0)
	**Muslim**	79 (9.4)	29 (36.7)	50 (63.3)	53 (67.1)	26 (32.9)
**Subtotal** **Total**		**844 (100)**	**329 (39.0)**	**515 (61.0)**	**673 (79.7)**	**171 (20.3)**

*Neg = Negative, Pos = Positive, IPIs = Intestinal Parasitic Infections

### Prevalence of intestinal parasitosis

The overall prevalence of intestinal parasitosis with a combination of FECT, STST, BCT, APC and RT-PCR was (79.7%;673/844) (Table 1). The distribution of intestinal parasitosis was higher, (86.7%;196/226) in the age group of 12–14 years, female participants (84.9%;370/436) and rural dwellers (80.1%;601/745) (Table 1).

Parasites identified among study participants were *S*. *stercoralis* (39.0%;329/844), hookworm species (33.2%;277/844), *Entamoeba histolytica/dispar* (23.8%;201/844), *Schistosoma mansoni* (20.4%;172/844), *Giardia duodenalis* (7.4%;62/844), *Ascaris lumbricoides* (4.5%;38/844), *Hymenolepis nana* (4.1%;35/844), *Enterobius vermicularis* (0.8%;7/844), *Trichuris trichiura* (0.7%;(6/844), *Taenia* spp. (0.5%;4/844) and *Fasciola* spp. (0.4%;3/844). Prevalence of single, double, triple, quadruple and quintuple intestinal parasitic infections among schoolchildren was (79.7%;673/844), (26.8%;226/844), (10.7%;90/844), (1.9%;16/844) and (0.1%;1/844), respectively (Table 2 and S1 Fig).

**Table 2:** Distribution of parasite species, single, double, triple, quadruple and quintuple infections among school children in Amhara Region, 2019.

Parasite species	Number of examined	Number	Percentage
***S*. *stercoralis***	844	329	39.0
**Hookworm species**	844	277	33.8
***S*. *mansoni***	844	172	20.4
***A*. *lumbricoides***	844	38	4.5
***H*. *nana***	844	35	4.1
***E*. *vermicularis***	844	7	0.8
***T*. *trichiura***	844	6	0.7
***Taenia species***	844	4	0.5
***F*. *hepatica***	844	3	0.4
***E*. *histolytical/dispar***	844	201	23.8
***G*. *duodenalis***	844	62	7.4
**Single infection**	844	673	79.7
**Double infections**	844	226	26.8
**Triple infections**	844	90	10.7
**Quadruple infections**	844	16	1.9
**Quintuple infections**	844	1	0.1

**Fig 3:**
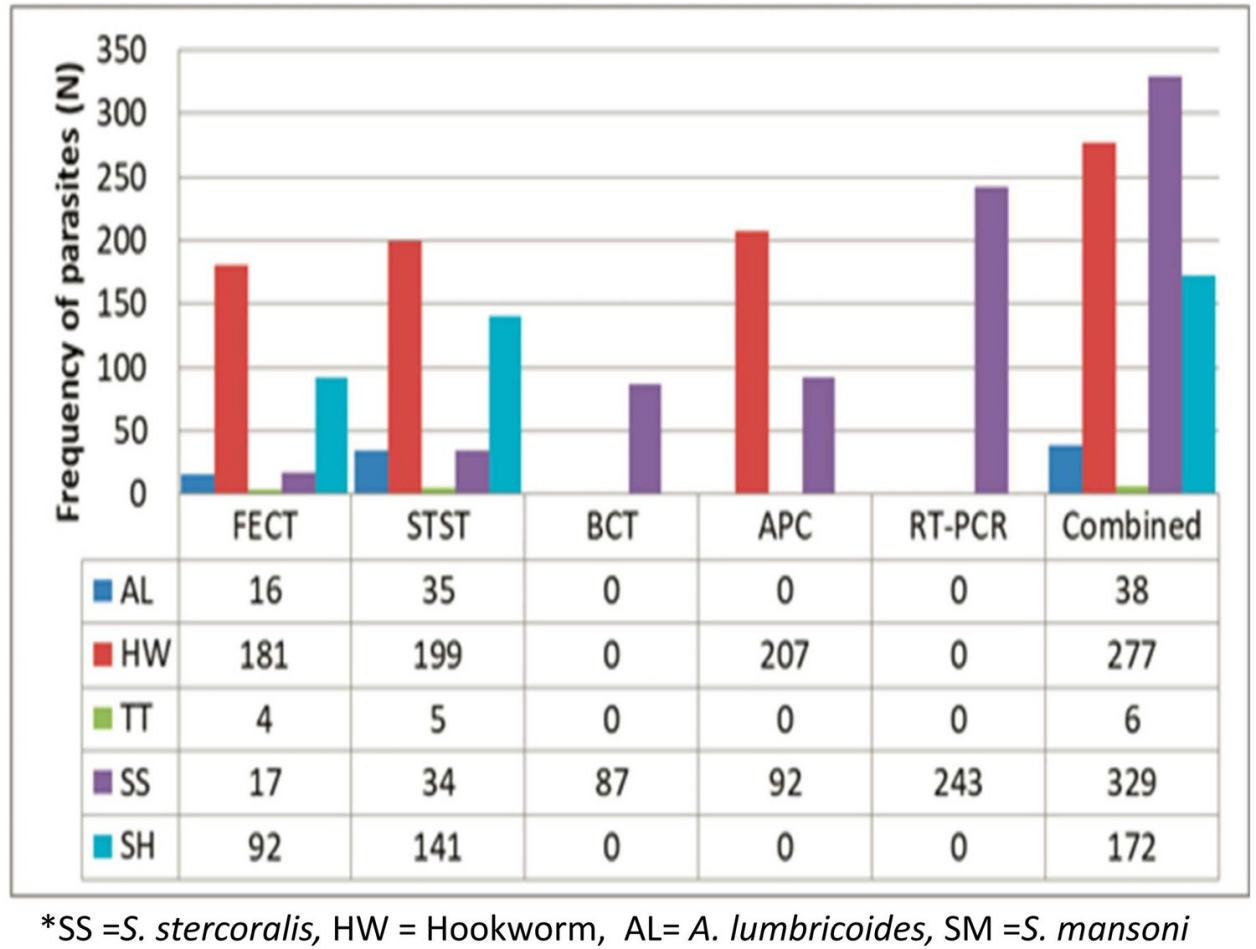
Detection frequency of intestinal parasites and *S*. *mansoni* by five different diagnostic methods from stool samples taken from schoolchildren in Amhara Region, 2019.

The prevalence of *S*. *stercoralis* by FECT, STST, BCT, APC, RT-PCR and combinations of the five methods were 17 (2.0%), 34 (4%), 87 (10.3%), 92 (10.9%), 243 (28.8%) and 329 (39.0%), respectively (Fig 3).

### Spatial distribution of soil-transmitted helminths and *S*. *stercoralis*

The overall prevalence of STHs among schoolchildren using a combination of parasitological techniques (FECT, STST, BCT and APC) was 38.0% (321/844). The highest distribution of STHs was recorded in highlands (49.4%; 82/166) followed by semi-highlands (40.1%; 218/543) and lowlands (15.6%: 21/135) areas. The prevalence rates of *A*. *lumbricoides* in highlands, hookworm in semi-highlands and *S*. *mansoni* in lowlands were 17.5% (29/166), 38.5% (209/543) and 34.1% (46/135), respectively (Table 3).

**Table 3:** Spatial distribution of STHs, *S*. *stercoralis* and *S*. *mansoni* among schoolchildren across altitudinal gradients in Amhara Region, 2019.

Cluster	Districts	Examined [N,%]	AL [N,%]	HW [N,%]	TT [N,%]	STHs [N,%]	SS [N,%]	SM [N,%]
**Highlands**	**Gozamen**	105 (16.0)	17 (16.2)	48 (45.7)	4 (3.8)	69 (65.7)	43 (41.0)	3 (2.9)
	**Tuledere**	61 (7.2)	12 (19.7)	0 (0)	1 (1.6)	13 (21.3)	24 (39.3)	0 (0)
**Sub-total**		**166 (19.7)**	**29 (17.5)**	**48 (28.9)**	**5 (3.0)**	**82 (49.4)**	**67 (40.4)**	**3 (1.8)**
**Semi-highlands**	**Bahir Dar**	276 (32.7)	6 (2.2)	160 (58.0)	1 (0.4)	167 (60.5)	136 (49.3)	79 (28.6)
	**Fogera**	159 (18.8)	0 (0)	27 (17.0)	0 (0)	27 (17.0)	52 (32.7)	42 (26.4)
	**Dangila**	108 (12.8)	2 (1.9)	22 (20.4)	0 (0)	24 (22.2)	39 (36.1)	2 (1.9)
**Sub-total**		**543 (64.3)**	**8 (1.4)**	**209 (38.5)**	**1 (0.2)**	**218 (40.1)**	**227 (41.8)**	**123 (22.6)**
**Lowlands**	**Jawi**	76 (9.0)	0 (0)	17 (22.4)	0 (0)	17 (22.4)	16 (21.1)	26 (34.2)
	**Metema**	59 (7.0)	1 (1.7)	3 (5.1)	0 (0)	4 (6.8)	19 (32.2)	20 (33.9)
**Sub-total**		**135 (16.0)**	**1 (0.7)**	**20 (14.8)**	**0 (0)**	**21 (15.6)**	**35 (25.9)**	**46 (34.1)**
**Overall total**		**844 (100)**	**38 (4.5)**	**277 (32.8)**	**6 (0.7)**	**321 (38.0)**	**329 (39.0)**	**172 (20.4)**

*AL = *A*. *lumbricoides*, HW = Hookworm, TT = *T*. *trichiura*, SS = *S*. *stercoralis*, SM = *S mansoni*, STH = Soil-transmitted Helminths, Pos = Positive

Prevalence of *S*. *stercoralis* in the highlands, semi-highlands and lowlands participants was (40.4%;67/166), (41.8%;227/543) and (25.9%;35/135), respectively (Table 3). The prevalence difference between altitudes was statistically significant (*p = 0*.*003*). Among the highlands, semi-highlands and lowlands areas, the highest prevalence of *S*. *stercoralis* was recorded (41%;43/105) in Guzamen (49.3%;136/276) in Bahir Dar Zuria and (32.2%;19/59) in Metema Districts, respectively (Table 3).

Prevalence of *S*. *stercoralis* co-infection with other parasites was 26.2% (221/844). The prevalence of single, double, triple and quadruple and quintuple infection of *S*. *stercoralis* with other parasites was (32.8%;108/329), (41.6%;137/329), (21.0%;69/329), (4.3%;14/329) and (0.1%;1/844), respectively. From the double infections, the prevalence of *S*. *stercoralis-*hookworm was the highest (43.8%; 60/137) (Table 4 and S2 Fig).

**Table 4:** The distribution of *S*. *stercoralis* co-infection with other intestinal parasites among schoolchildren in Amhara Region, 2019.

Parasites identified	Total examined	Number	Percentage
**SS**	844	108	12.8
**SS+AL**	844	9	1.1
**SS+AM**	844	33	3.9
**SS+GD**	844	7	0.8
**SS+HN**	844	5	0.6
**SS+HW**	88	60	7.1
**SS+SM**	844	21	2.5
**SS+TT**	844	2	0.2
**SS+AL+AM**	844	1	0.1
**SS +AL+GD**	844	1	0.1
**SS+AL+TT**	844	1	0.1
**SS+AM+TT**	844	1	0.1
**SS+HW+SM**	844	19	2.3
**SS+SM+AM**	844	8	1.0
**SS+HW+AM**	844	23	2.7
**SS+HW+GD**	844	4	0.5
**SS+HW+HN**	844	1	0.1
**SS+FH+AM**	844	1	0.1
**SS+AM+GD**	844	7	0.8
**SS+SM+GD**	844	2	0.2
**SS** +**HW+AL+AM**	844	1	0.1
**SS+HW+AL+EV**	844	2	0.2
**SS+HW+GD+AM**	844	1	0.1
**SS+HW+SM+AM**	844	6	0.7
**SS+HW+SM+GD**	844	2	0.2
**SS+HW+SM+HN**	844	1	0.1
**SS+SM+HN+AM**	844	1	0.1
**SS+HW+SM+GD+AM**	844	1	0.1
**Total**	**844**	**329**	**39.0%**

*SS = *S*. *stercoralis*, HW = Hookworm, AL = A. *lumbricoides*, AM = *E*. *histolytica/dispar*, GD = *G*. *duodenalis*, SM = *S*. *mansoni*, HN = *H*. *nana*, TT = *T*. *trichiura*, FH = *Fasciola hepatica*

### Clinical symptoms associated with *Strongyloides stercoralis* infection

Schoolchildren who had abdominal pain were 2.44 times (AOR = 2.44; 95% CI:1.65–3.72) more likely to be infected with *S*. *stercoralis* parasites compared with schoolchildren without abdominal pain. Similarly, schoolchildren who had a cough were 1.63 times (AOR = 1.63;95% CI:1.09–2.42) more likely to be infected by *S*. *stercoralis* than those children without cough. Likewise, schoolchildren with urticaria (a type of skin rash) were 2.49 times (AOR = 2.49;95%CI:1.50–4.01) more infected by *S*. *stercoralis* parasite than those schoolchildren who did not have skin rash. The odds of being infected by *S*. *stercoralis* in malnourished schoolchildren were 1.49 times (AOR = 1.49;95%:1.10–2.01) higher compared with well-nourished (normal) children (Table 5).

**Table 5:** Univariate and multivariate analyses of clinical symptoms associated with *S*. *stercoralis* infection among schoolchildren in Amhara Region, 2019.

Variables		*Number*		*COR (95%CI)*	*P-value*	*AOR (95%CI)*	*P-value*
		*Pos*	*Neg*				
Abdominal pain	Yes	*104*	*81*	*2*.*48 [1*.*78–3*.*45]*	*0*.*000*	*2*.*48 [1*.*65–3*.*72]*	*0*.000*
	No	225	434	1			
Diarrhea	Yes	90	101	1.54 [1.12–2.14]	0.009	1.17 [0.77–1.79]	0.469
	No	239	414	1			
Loss of appetite	Yes	97	117	1.42 [1.04–1.95]	0.028	1.1.02 [0.68–1.54]	0.923
	No	232	398	1			
Feel tiredness	Yes	95	145	1.04 [0.76–1.41]	0..821		
	No	234	370	1			
Presence of cough	Yes	96	129	1.23 [0.90–1.68]	0.186	1.63 [1.09–2.42]	0.017*
	No	233	386	1			
Presence of urticaria	Yes	67	45	2.67 [1.78–4.01]	0.000	2.49 [1.50–4.13]	0.000*
	No	262	470	1			
Stool pass per-day	3–5	100	154	1.02 [0.76–1.38]	0.879		
	1–2	229	361	1			
BMI	Nor	195	354	1			
	Mal	134	161	1.51 [1.13–2.02]	0.005	1.49 [1.10–2.01]	0.009*

*Pos = Positive, Neg = Negative, BMI = Body Mass Index, COR = Crude Odds Ratio, AOR = Adjusted Odds Ratio, Nor = Normal, Mal = Malnourished

## Discussion

Soil-transmitted helminths and *Schistosoma* spp. infections are common causes of morbidity among children in the tropics and subtropics [27]. STHs prevalence (38.0%) is similar to 38.3% previously reported from Gena Bossa Woreda, Ethiopia [28], and 39.0% in Bahir Dar Zuria woreda, northwest Ethiopia [29]. Similarly, the prevalence of *S*. *mansoni* (20.4%) was recorded in this study. This result is lower than what were previously reported from southwest Ethiopia (28.7%) [30], and western Ethiopia (53.9%) [31]. The difference in schistosomiasis prevalence might be due to the variation in the endemicity of *S*. *mansoni* and frequency of cercaria-infected water body contact. Multiple intestinal parasitic infection were high in the present study. This finding is similar with previous reports [32,33].

*Strongyloides stercoralis* infection is one of the most common parasitic infections among children in the tropics and subtropics [11]. The prevalence of *S*. *stercoralis* among schoolchildren was 39.0%. Even though this result is slightly lower than the 48.6% previously reported from rural district of Bahir Dar [7], it is higher than earlier reports in Africa, e.g., 20.7% from rural Bahir Dar [17], 21.4% from western Angola [34], 31.6% 27% from rural Côte d’lvoire [35], and 12.8% from Angola [36], being one of the highest prevalence in sub-Saharan Africa, to our knowledge. The variations might be due to differences in the sample size, the diagnostic methods used, the amount and number of stool samples taken, and geographical area differences. Generally, unless we are applying a “Gold” standard diagnostic method for *S*. *stercoralis* infection, underdiagnosing and under-reporting of strongyloidiasis continues to be a problem in endemic areas. A combination of diagnostic methods gives a better detection rate than using a single method to report the true prevalence of *S*. *stercoralis* infection.

The prevalence of *S*. *stercoralis* infection was increased as the age of children increased, which is similar to what were previously reported in similar studies from Lao *People’s Democratic Republic* [37] and rural Cambodia [12] and again in Africa [7]. This might be associated with more outdoor activities of older schoolchildren that would have probably increased the exposure rate. Moreover, because of the autoinfective life cycle, most children infected with *S*. *stercoralis* become chronic carriers in the absence of ivermectin treatment.

*Strongyloides stercoralis* prevalence was higher in male schoolchildren (45.0%) than in female schoolchildren (32.6%), which is similar to a previous report [12]. Similarly, a higher prevalence of *S*. *stercoralis* was observed in children from rural areas than urban areas. This result is consistent with earlier reports [38,39]. This might be explained by the fact that a higher number of male children compared to females are engaged in outdoor activities like agriculture, irrigation activities (“khat” and rice growing), cattle keeping, and playing with soil.

The prevalence of *S*. *stercoralis* (40.4%) obtained in the highlands areas in the current study is higher than the 18.6% reported from Peru [40]. Even though the higher prevalence of the infection in highland areas of the present study might be due to the employment of more sensitive methods and/or the combined use of five diagnostic methods, this finding is surprising. This is because the prevalence of STHs diminishes as the altitude increases. In the semi-highland area, *S*. *stercoralis* prevalence was 41.8% which is lower than the 48.6% reported from a rural community of Ethiopia [7], but it is higher than the 20.7% previously reported from northwest Ethiopia [17]. Generally, higher prevalence of *S*. *stercoralis* observed in semi-highlands than in highlands or lowlands might be due to the presence of ambient soil temperature and wet environment for the existence of larvae. Prevalence of *S*. *stercoralis* was the lowest (25.9%) in lowland areas as compared to the highland and semi-highland areas. But this current report is higher than the 11.1% previously reported from the lowland areas of southern Ethiopia [41], but it is comparable with 26.4% reported from Peru [40]. A possible explanation for the discrepancy might be the presence of wet, ambient environmental factors for larval development, age of the study participants, and diagnostic methods. Alternatively, it might be due to age differences in study participants; the majority of the participants (74.5%) from the Peru report are being from the lowland area in the age range 13–52 year.

Most *S*. *stercoralis* infections are asymptomatic; however, there are some clinical symptoms seen in *S*. *stercoralis*-infected persons [42]. Schoolchildren who had abdominal pain (*p<0*.*001*) and cough were significantly associated (*p = 0*.*011*) with *S*. *stercoralis* infection. These findings are similar to previous results obtained in Preah Vihear Province, Cambodia [43], where they showed the presence of the *S*. *stercoralis* larvae in the lungs and the *S*. *stercoralis* adult in the intestine, respectively. In the lungs allergic reaction may occur due to the migration of the larvae (Loeffler’s syndrome) that leads to cough. In the intestine, it is likely that a combination of different immune reactions to the parasite as well as the pathology caused by migrating larvae through the tissues that might cause abdominal pain [44].

Skin rash, either due to “larva currens” or a systemic allergic response leads to a generalized urticaria. It is one of the clinical signs and symptoms of individuals infected with *S*. *stercoralis* [45]. Schoolchildren who had urticaria were significantly associated (*p = 0*.*003*) with *S*. *stercoralis* infection. This finding has been previously described in people infected with *S*. *stercoralis* [46–48]. This could be explained by the fact that migration of the *S*. *stercoralis* larvae under the skin possibly induces an immunological reaction leading to larva currens or/and urticaria on the skin. Generalized pruritus, prurigo, maculopapular exanthema may also be seen with naked eyes.

Those schoolchildren who had BMI<18.5 were significantly associated (*p = 0*.*017*) with *S*. *stercoralis* infection, which is consistent with the previous report from Cambodia [46]. This could be explained by the autoinfection cycle, or the parasitic female *S*. *stercoralis* continuing to lay eggs leading to the infection of the small intestine by the larvae which results in subsequent thickening of the wall of intestine and causing intestinal oedema, inflammation and atrophy of the villi, thereby resulting in poor intestinal absorption [49].

Finally, the use of a single sample per patient, because of logistic problem, was a limitation of the study. Increasing the number of stool samples per patient will allow more number of *S*. *stercoralis* numbers to be detected. It is also interesting to compare the sensitivity of a SYBR-Green RT-PCR format with a Taqman RT-PCR in order to have a more precise data on the sensitivity of both assays and this should be considered in the future studies.

## Conclusion

Prevalence of *S*. *stercoralis* was high and the distribution of *S*. *stercoralis* varied across altitude gradients in Amhara Region. Clinical symptoms including abdominal pain, cough, skin rash and malnourishment were significantly associated with *S*. *stercoralis* infection. Therefore, significant attention should be given to *S*. *stercoralis* infection for both its proper diagnosis using a more sensitive diagnostic approach and treatment using ivermectin in STHs control programs to prevent *S*. *stercoralis* infection in Amhara Region. The role of other environmental factors that facilitate the existence and transmission of *S*. *stercorali*s should also be further investigated.

## Supporting information

S1 Fig Distribution of parasite infections among school children in Amhara Region. (TIF)

S2 Fig Distribution of *S*. *stercoralis* co-infection with other parasites in Amhara Region. (TIF)
