# Supplementary figures and images for: Prevalence of Strongyloides stercoralis infection and associated clinical symptoms among schoolchildren living in different altitudes of Amhara National Regional State, northwest Ethiopia
Source: PLoS Negl Trop Dis. 2022 Apr 28;16(4):e0010299. doi: 10.1371/journal.pntd.0010299 (PMC9049318; doi:10.1371/journal.pntd.0010299)

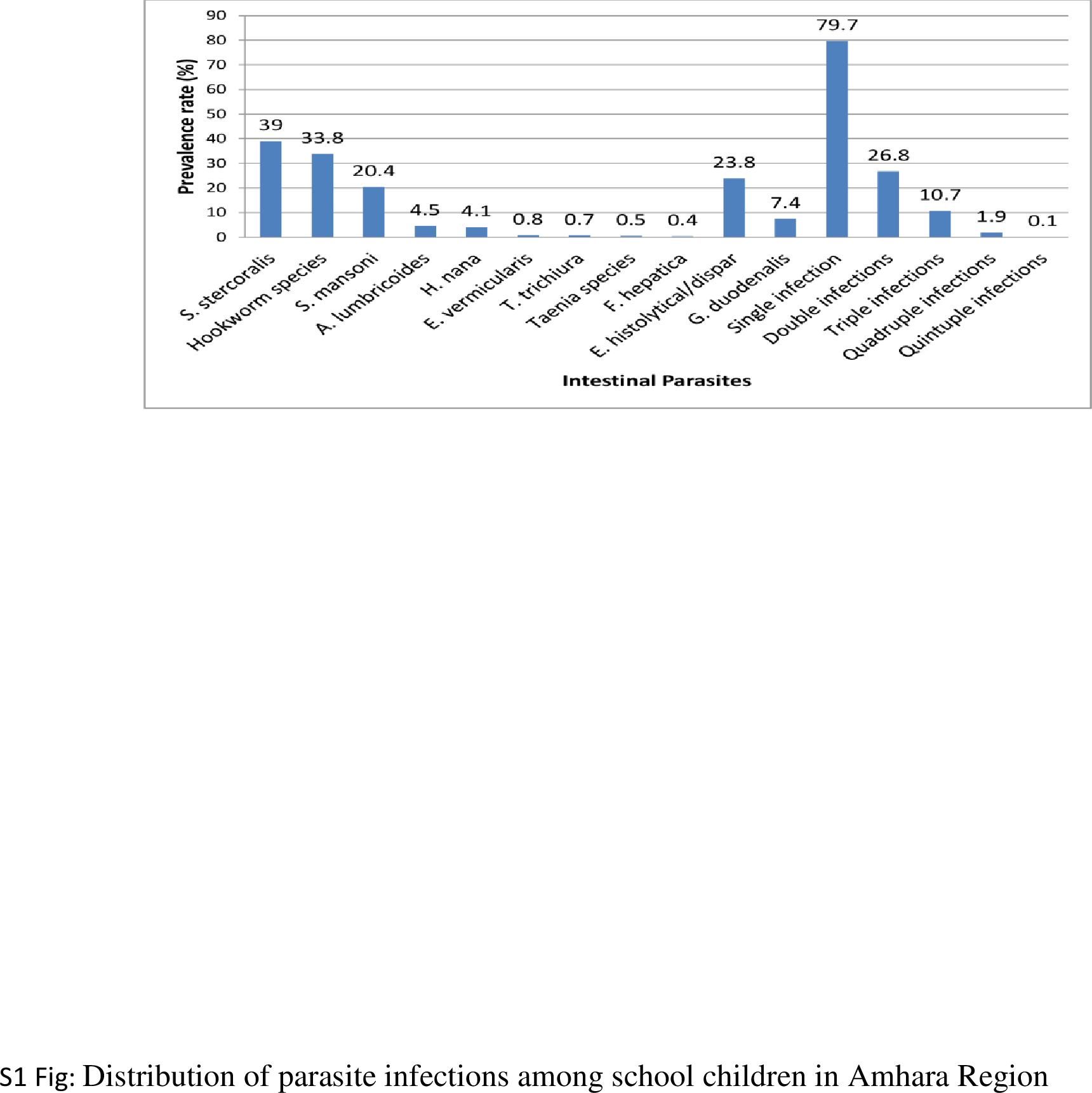

Supplement: S1 Fig — (TIF) [file pntd.0010299.s001.tif]

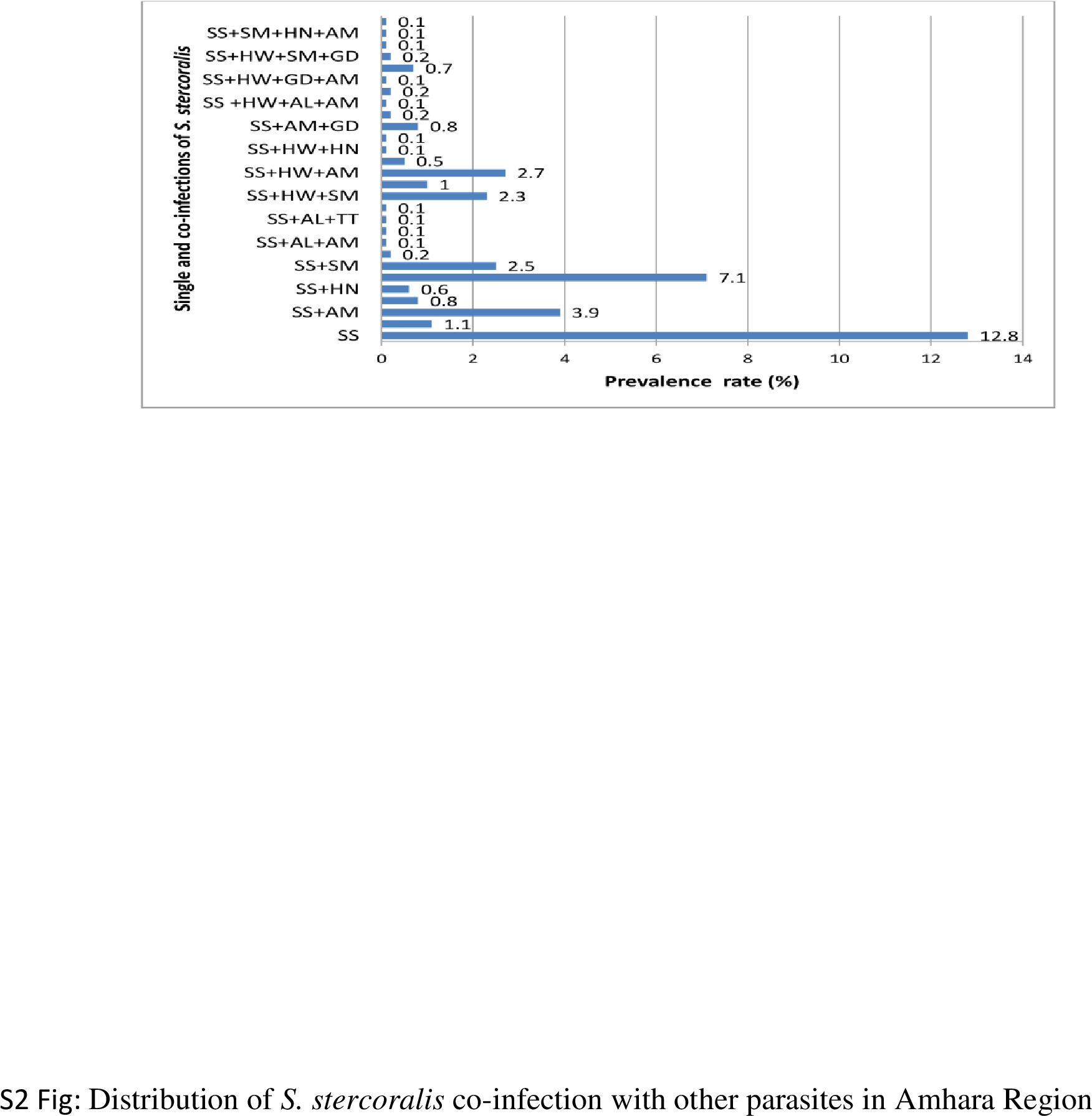

Supplement: S2 Fig — (TIF) [file pntd.0010299.s002.tif]
